# Site-Specific Survival and Complications of Extra-oral Craniofacial Implants in Irradiated and Non-irradiated Patients: A Systematic Review and Meta-Analysis

**DOI:** 10.7759/cureus.117428

**Published:** 2026-10-04

**Authors:** V Srishti, Sanjeev Singh, Saumya Sharma, Gulab C Baid, Priyabrata Jena

**Affiliations:** 1 Department of Prosthodontics, Crown, Bridge and Implantology, Maitri College of Dentistry and Research Centre, Durg, IND

**Keywords:** auricular implants, craniofacial implants, implant survival, meta-analysis, orbital implants, radiotherapy, systematic review

## Abstract

Extra-oral craniofacial implants are employed in order to keep facial prostheses in place in people who have craniofacial defects. The rate at which implants survive can differ according to the anatomical site and whether or not the patient has had radiotherapy. This systematic review and meta-analysis looked at the survival rates and complications according to site in both irradiated and non-irradiated patients. A PRISMA-based search of PubMed/MEDLINE, Scopus, Web of Science, and the Cochrane Library (from 2000 to 2025) resulted in 15 studies being selected for qualitative synthesis, eight for the primary meta-analysis, and five for the secondary meta-analysis. Auricular implants had a better survival rate than orbital implants (RR = 2.64; 95% CI: 1.32-5.30; p = 0.006). Radiotherapy was found to increase the risk of implant failure (RR = 1.99; 95% CI: 1.09-3.66; p = 0.03). The most common complications were reactions of the soft tissues around the implant, skin inflammation, the need for prosthetic maintenance, and loss of the implant. On the basis of the current evidence, auricular implants show the most favorable survival, while radiotherapy has a negative effect on the outcomes. When treating patients, both the anatomical site and their radiation history should be taken into account for planning of treatment and for follow-up.

## Introduction and background

Craniofacial abnormalities resulting from congenital anomalies, trauma, infection, or following tumor surgery lead to considerable functional, aesthetic, and psychosocial difficulties and have a negative impact on quality of life and on social reintegration [1,2]. Rehabilitation is still a difficult problem in the field of maxillofacial prosthodontics since it is necessary to restore facial form, retention, stability, and patient acceptance [1,2]. Although adhesive-retained prostheses have been used in the traditional way, limitations including poor retention, displacement, skin irritation, and degradation of the adhesive have resulted in the adoption of implant-retained rehabilitation [3].

When Brånemark introduced osseointegration, he laid the biological foundation for direct integration between bone and titanium [4,5]. Clinical studies have shown that osseointegrated implants can be successfully used in auricular, orbital, nasal, and midfacial prostheses [3,6-10]. Today, implants used with prostheses represent a reliable method that enhances retention, stability, aesthetics, and patient satisfaction [7-11].

The success of an implant is influenced by the site at which it is placed, the quality of the bone, the surgical technique used, the design of the prosthesis, and various patient-related factors. The anatomical location is a key factor since implants in the ear area yield good results because of the presence of cortical bone and good blood supply, while those placed in the orbital and midfacial areas are usually situated in areas that are compromised and have a higher risk of failure [7,9-17].

Radiotherapy is also a crucial element; radiation causing hypovascularity, hypocellularity, and impaired bone remodeling hinders osseointegration and stability [18,19]. Although some studies have found a reduced survival rate among irradiated patients [13-17,20], other studies indicate that acceptable results can be achieved using appropriate protocols [19,20].

Systematic reviews carried out in the past have examined the effects of radiotherapy on implant survival [21-25], but many relied on early studies with outdated implant designs, surgical techniques, and radiotherapy methods. Importantly, most did not provide a comprehensive site‑specific analysis of auricular, orbital, and nasal implants in the context of radiation exposure. This lack of detailed, contemporary evidence highlights a clear knowledge gap, which our review aims to address through an updated synthesis of studies published after 2000.

This systematic review and meta-analysis was carried out with the aim of assessing the site-specific survival rates and complications associated with extraoral craniofacial implants in both irradiated and non-irradiated patients, using only the studies that had been published from 2000 onwards.

## Review

Methodology

Protocol and Reporting Guidelines

This systematic review and meta-analysis was conducted in accordance with the Preferred Reporting Items for Systematic Reviews and Meta-Analyses (PRISMA) 2020 statement [26]. The review protocol was retrospectively registered with the Open Science Framework (OSF Registries) on September 10, 2026 (registration DOI: 10.17605/OSF.IO/T3PZ9; https://osf.io/k8w74).

Focused Question

The review used the PICOS (population, intervention, comparison, outcomes, and study) framework to ask: What are the site-specific survival rates and complications associated with extraoral craniofacial implants, and in what way does radiotherapy affect the outcomes of patients who have received it as compared to those who have not? The population included patients having extraoral craniofacial implants; the intervention was the placement of the implant by anatomical site; the comparison was between those who had radiotherapy and those who had not; the outcomes were survival and complications; the study design was that of observational studies. The anatomical site and radiotherapy status were predetermined subgroups, and the complications were qualitatively synthesized when pooling could not be carried out.

Eligibility Criteria

For inclusion, we required human clinical studies carried out between 2000 and 2025 that reported on survival, failure, or complications of extraoral craniofacial implants and had a follow-up period of at least 12 months; for the subgroup analysis, radiotherapy status was necessary, but it was not required for the site-specific analysis. Studies excluded were those involving animals or conducted in vitro, case reports, case series with fewer than five patients, reviews, conference abstracts, editorials, and studies which lacked outcome data. Recent studies were excluded only if they did not meet the eligibility criteria (e.g., insufficient follow‑up, lack of site‑specific data, or incomplete reporting.

Search Strategy

The databases consulted were PubMed/MEDLINE, Scopus, Web of Science, and the Cochrane Library; the reference lists were examined manually. The search terms used were 'extra oral implants', 'craniofacial implants', 'facial implants', 'implant survival/failure/complications', 'auricular/orbital/nasal implants', and 'radiotherapy/irradiated patients'. Only English-language publications were included; grey literature was not considered. The complete search strings for each database, including Boolean operators, medical subject headings (MeSH) terms, and filters, are provided in the Appendix.

Study Selection

The titles and abstracts were screened, and then the full texts were reviewed in accordance with the inclusion criteria. Any disagreements were settled by consensus.

Data Extraction

A standard form was used to record the author, the year, the design, the sample size, the implant site and the number, the follow-up period, the survival or failure results, the radiotherapy status, and the complications. The numerical data were checked before being analysed.

Quality Assessment

The methodological quality was evaluated by means of the Newcastle-Ottawa Scale (NOS) [27].

Statistical Analysis

A meta-analysis was carried out using RevMan (version 5.4; Cochrane Collaboration, London, UK), and risk ratios (RRs) together with 95% confidence intervals (CIs) were calculated for the dichotomous outcomes. A random‑effects model was used to account for clinical and methodological variability across studies. Heterogeneity was evaluated by means of I², and if this was greater than 50%, it was considered to indicate substantial heterogeneity. A probability value of less than 0.05 was taken to indicate statistical significance.

Results

Study Selection

The search carried out electronically yielded 412 records. Duplicates were removed, and the titles and abstracts were screened before the full-text articles were evaluated for eligibility. Fifteen of the studies were included in the qualitative synthesis. Eight studies were included in the primary meta-analysis of site-specific survival and five in the secondary meta-analysis of radiotherapy effects. The process of selection is illustrated in the PRISMA flow diagram (Figure 1).

**Figure 1:**
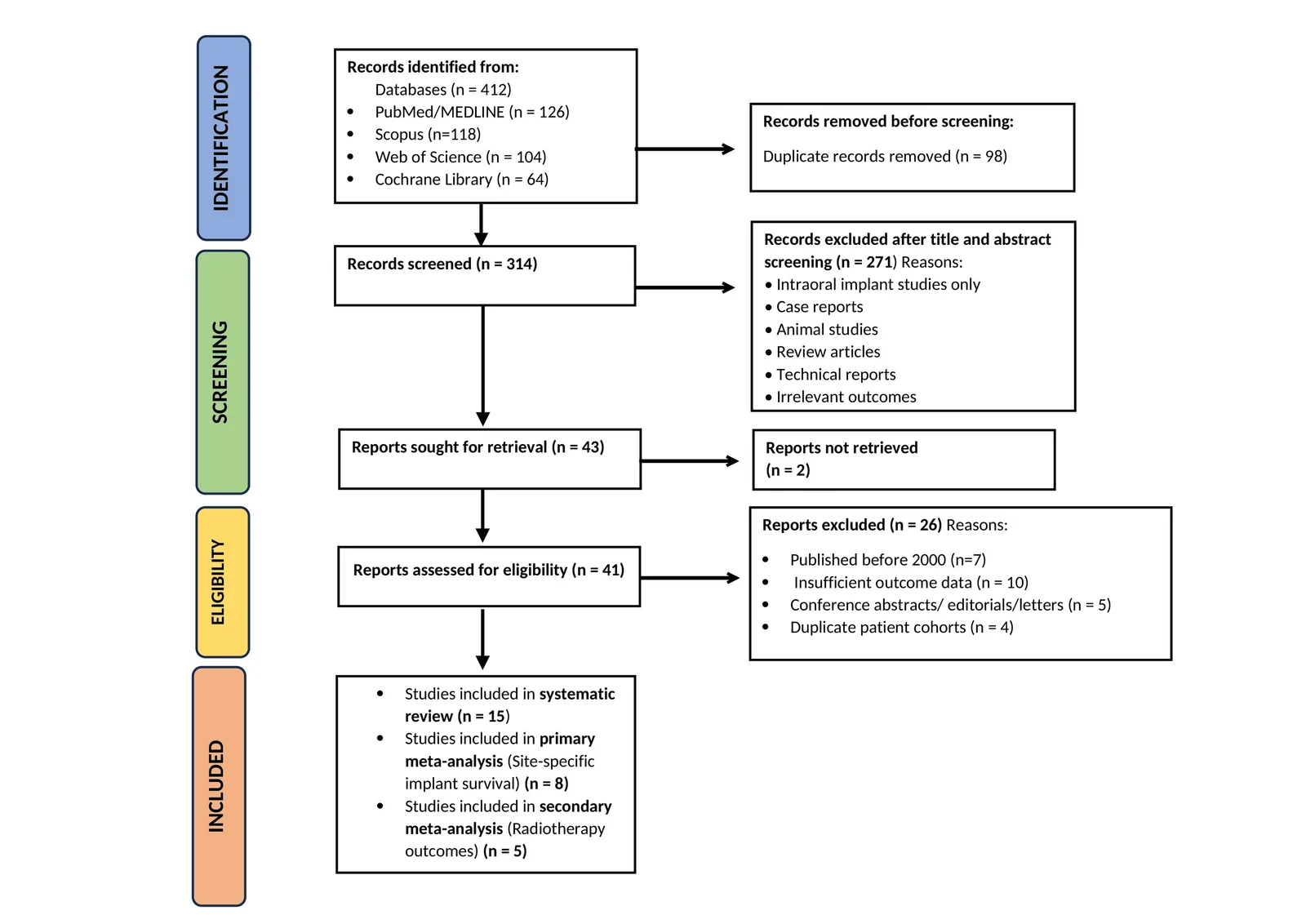
PRISMA 2020 flow diagram illustrating the identification, screening, eligibility assessment, and inclusion of studies in the systematic review and meta-analysis PRISMA: Preferred Reporting Items for Systematic reviews and Meta-Analyses Fifteen studies were included in the qualitative synthesis (systematic review). Out of those 15, eight studies met the criteria for inclusion in the primary meta‑analysis (site‑specific implant survival). Out of the same 15, five studies met the criteria for inclusion in the secondary meta‑analysis (radiotherapy outcomes).

Study Characteristics

Most studies were retrospective and evaluated auricular, orbital, nasal, and midfacial implants. Follow‑up ranged from nearly three years to more than 15 years. Detailed characteristics are presented in Table 1.

**Table 1:** Characteristics of the included studies

Author	Year	Study Design	Implant Site	Total Implants	Follow-Up
Schoen et al. [9]	2001	Retrospective	Auricular, orbital	75	45 months
Abu-Serriah et al. [10]	2001	Retrospective	Auricular, orbital, nasal	141	Long term
Roumanas et al. [11]	2002	Retrospective	Auricular, orbital, nasal	207	Up to 14 years
Scolozzi et al. [12]	2004	Retrospective	Orbital, orbitonasal, orbitonasomaxillary, nasal	62	2.7 years
Granstrom et al. [13]	2005	Retrospective	Auricular, orbital, nasal, maxillofacial	631	Mean 6.3 years
Toljanic et al. [14]	2005	Multicentre retrospective	Orbital	153	52.6 months
Visser et al. [15]	2008	Retrospective	Auricular, orbital, nasal	270	88 months
Karayazgan-Saracoglu et al. [16]	2010	Retrospective	Auricular, orbital, nasal, midfacial	159	Up to 84 months
Curi et al. [17]	2012	Retrospective	Auricular, orbital, nasal, midfacial	150	48.1 months
Oliveira et al. [18]	2013	Retrospective	Auricular, orbital, nasal	164	Variable
Mueller et al. [19]	2015	Retrospective	Midfacial, orbital	36	Mean 43 months
Woods and Chandu [20]	2017	Retrospective	Auricular, orbital, nasal	156	10 years
Dos Reis et al. [21]	2017	Retrospective	Auricular, orbital, nasal	138	11-111 months
Subramaniam et al. [22]	2018	Retrospective cohort	Auricular, orbital, nasal	341	Long term
Alberga et al. [23]	2022	Retrospective cohort	Auricular, orbital, nasal, midfacial	525	Long term

Quality Assessment

NOS scores indicated moderate-to-good methodological quality. No study was excluded based on quality (Table 2).

**Table 2:** Newcastle-Ottawa Scale (NOS) quality assessment of the included studies

Study	Selection	Comparability	Outcome	Total Score	Quality
Schoen et al. [9], 2001	4	1	3	8	Good
Abu-Serriah et al. [10], 2001	4	1	3	8	Good
Roumanas et al. [11], 2002	3	1	2	6	Moderate
Scolozzi et al. [12], 2004	3	1	3	7	Good
Granstrom et al. [13], 2005	3	1	2	6	Moderate
Toljanic et al. [14], 2005	3	1	2	6	Moderate
Visser et al. [15], 2008	4	1	3	8	Good
Karayazgan-Saracoglu et al. [16], 2010	3	1	3	7	Good
Curi et al. [17], 2012	4	1	3	8	Good
Oliveira et al. [18], 2013	4	2	3	9	Good
Mueller et al. [19], 2015	3	1	2	6	Moderate
Woods and Chandu [20], 2017	4	1	3	8	Good
Dos Reis et al. [21], 2017	3	1	2	6	Moderate
Subramaniam et al. [22], 2018	4	1	3	8	Good
Alberga et al. [23], 2022	4	2	3	9	Good

Implant Survival and Complications

Implant survival was defined as the continued presence of a functioning implant at last follow‑up. Implant failure was defined as loss or removal of the implant due to lack of osseointegration or complications. Complications included peri‑implant soft tissue reactions, inflammation, granulation tissue, or prosthetic maintenance issues. Survival varied by site. Auricular implants consistently showed the highest survival, while orbital implants had lower survival and higher failure rates [10-23]. Reported complications included peri‑implant soft tissue reactions, skin inflammation, granulation tissue, prosthetic maintenance, and implant loss [11-23]. Several studies noted reduced survival in irradiated patients [13,14,18,22,23]. Outcomes are summarized in Table 3.

**Table 3:** Survival outcomes and reported complications of extra-oral craniofacial implants

Author	Implant Site	Survival Rate	Major Findings/Complications
Schoen et al. [9], 2001	Auricular, Orbital	93.3% overall; 100% non-irradiated; 87.8% irradiated	Implant survival significantly lower in irradiated bone; no osteoradionecrosis observed; peri-implant tissues generally healthy.
Abu-Serriah et al. [10], 2001	Auricular, Orbital, Nasal	Site-specific survival varied according to anatomical location	Higher implant failure rates observed in orbital and nasal sites compared with auricular sites
Roumanas et al. [11], 2002	Auricular, Orbital, Nasal	89% overall	Continuous implant loss observed in orbital sites; prosthetic maintenance frequently required
Scolozzi et al. [12], 2004	Orbital, Orbitonasal, Orbitonasomaxillary, Nasal	100%	No implant failures reported; mild peri-implant skin reactions managed with hygiene measures
Granstrom et al. [13], 2005	Auricular, Orbital, Nasal, Maxillofacial	Higher implant survival following hyperbaric oxygen therapy	Radiation-related implant loss and peri-implant soft tissue complications
Toljanic et al. [14], 2005	Orbital	73.2% cumulative survival	No significant effect of radiotherapy, implant location, or hyperbaric oxygen therapy on implant survival
Visser et al. [15], 2008	Auricular, Orbital, Nasal	88.8% overall; 78.8% irradiated; 95.2% non-irradiated	Severe soft tissue reactions were more frequent in orbital defects
Karayazgan-Saracoglu et al. [16], 2010	Auricular, Orbital, Nasal, Midfacial	91.4% overall	Peri-implant soft tissue reactions were the most common complication
Curi et al. [17], 2012	Auricular, Orbital, Nasal, Midfacial	Auricular 94.1%; nasal 90.9%; orbital 100%; midfacial 100%	One case of osteoradionecrosis; most implant failures occurred before loading
Oliveira et al. [18], 2013	Auricular, Orbital, Nasal	95.3% irradiated; 95.1% non-irradiated	Failed osseointegration was the primary cause of implant loss
Mueller et al. [19], 2015	Midfacial, Orbital	80.6%	Seven implant failures occurred in reconstructed midfacial defects
Woods and Chandu [20], 2017	Auricular, Orbital, Nasal	72.4% overall	Orbital implants demonstrated the highest failure rates
Dos Reis et al. [21], 2017	Auricular, Orbital, Nasal	Auricular 92%; orbital 95.9%; nasal 92.9%	Implant survival differed according to implant site
Subramaniam et al. [22], 2018	Auricular, Orbital, Nasal	Lower survival in irradiated patients	Radiotherapy was associated with increased implant loss and peri-implant soft tissue complications
Alberga et al. [23], 2022	Auricular, Orbital, Nasal, Midfacial	Favorable long-term implant survival across craniofacial sites	Implant loss was associated with oncologic treatment and radiotherapy exposure

Orbital Versus Auricular Implants

Eight studies [10,15,17,18,20-23] showed orbital implants had a significantly higher risk of failure than auricular implants (RR = 2.64; 95% CI: 1.32-5.30; p = 0.006; I² = 70%) (Figure 2).

**Figure 2:**
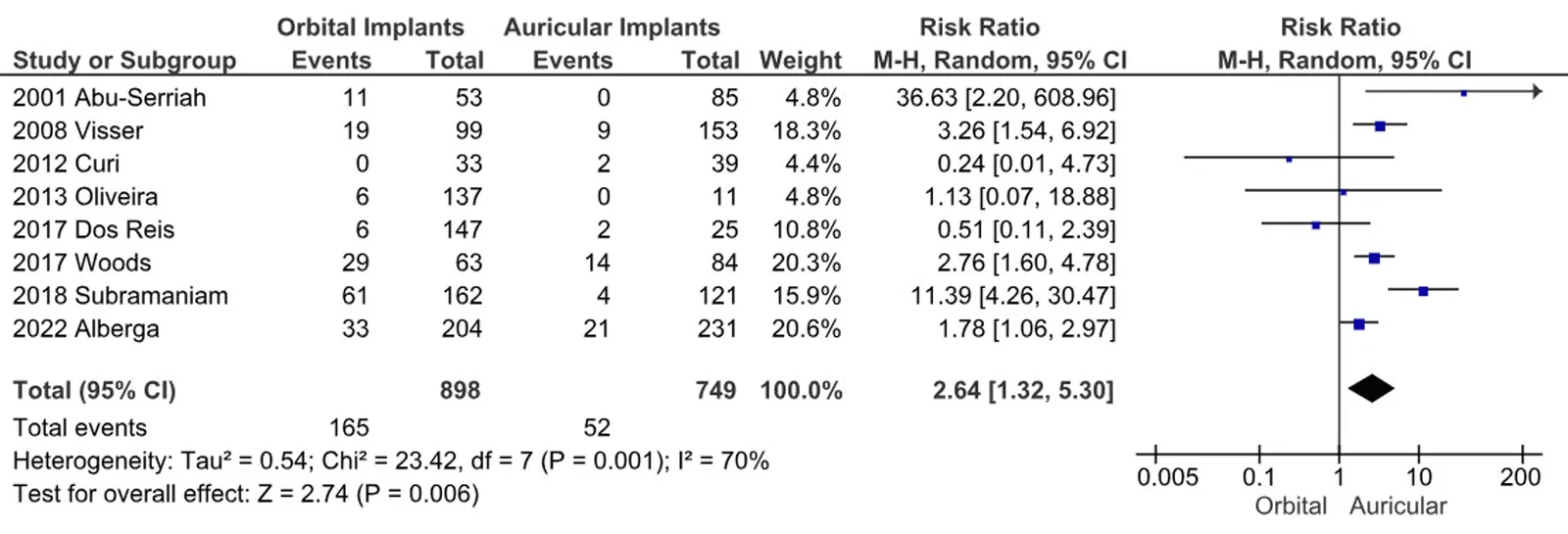
Forest plot showing the comparison of implant failure risk between orbital and auricular craniofacial implants Source: [10,15,17,18,20-23]

Nasal Versus Auricular Implants

Eight studies [10,15,17,18,20-23] found no significant difference between nasal and auricular implants (RR = 1.77; 95% CI: 0.83-3.80; p = 0.14; I² = 35%) (Figure 3). Several individual studies demonstrated wide confidence intervals, reflecting imprecision due to small sample sizes, and this limitation should be acknowledged when interpreting pooled results.

**Figure 3:**
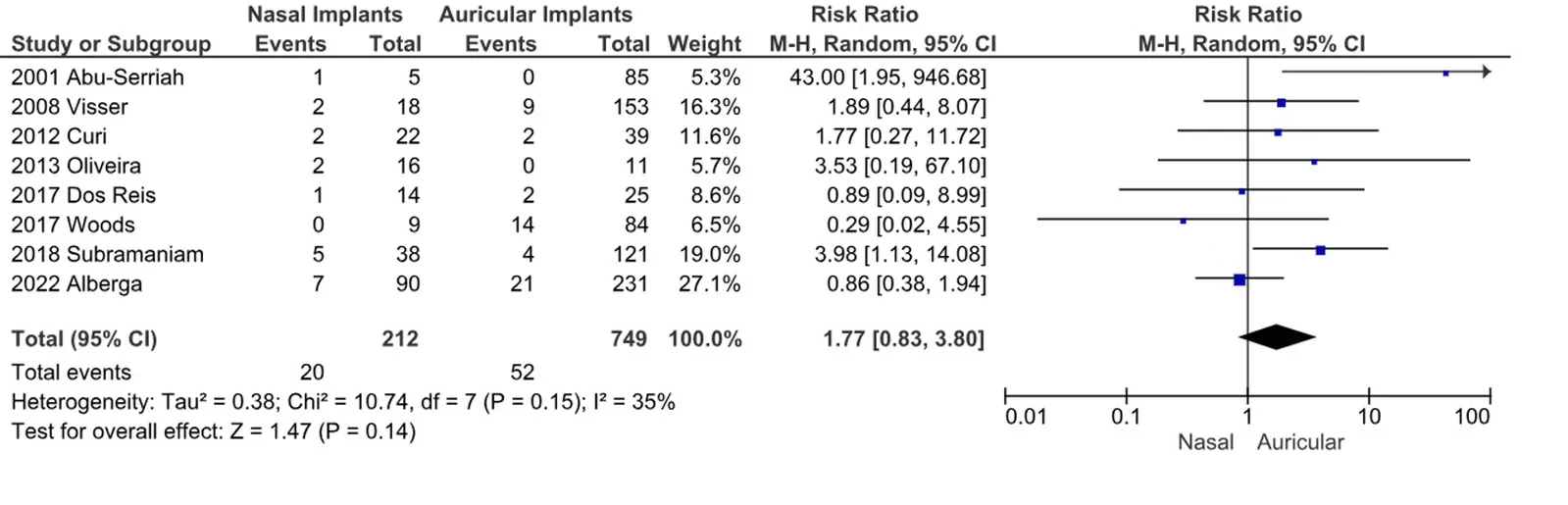
Forest plot showing the comparison of implant failure risk between nasal and auricular craniofacial implants Source: [10,15,17,18,20-23]

Nasal Versus Orbital Implants

Eight studies [10,15,17,18,20-23] reported no significant difference between nasal and orbital implants (RR = 0.71; 95% CI: 0.36-1.39; p = 0.32; I² = 41%) (Figure 4). Several individual studies demonstrated wide confidence intervals, reflecting imprecision due to small sample sizes, and this limitation should be acknowledged when interpreting pooled results.

**Figure 4:**
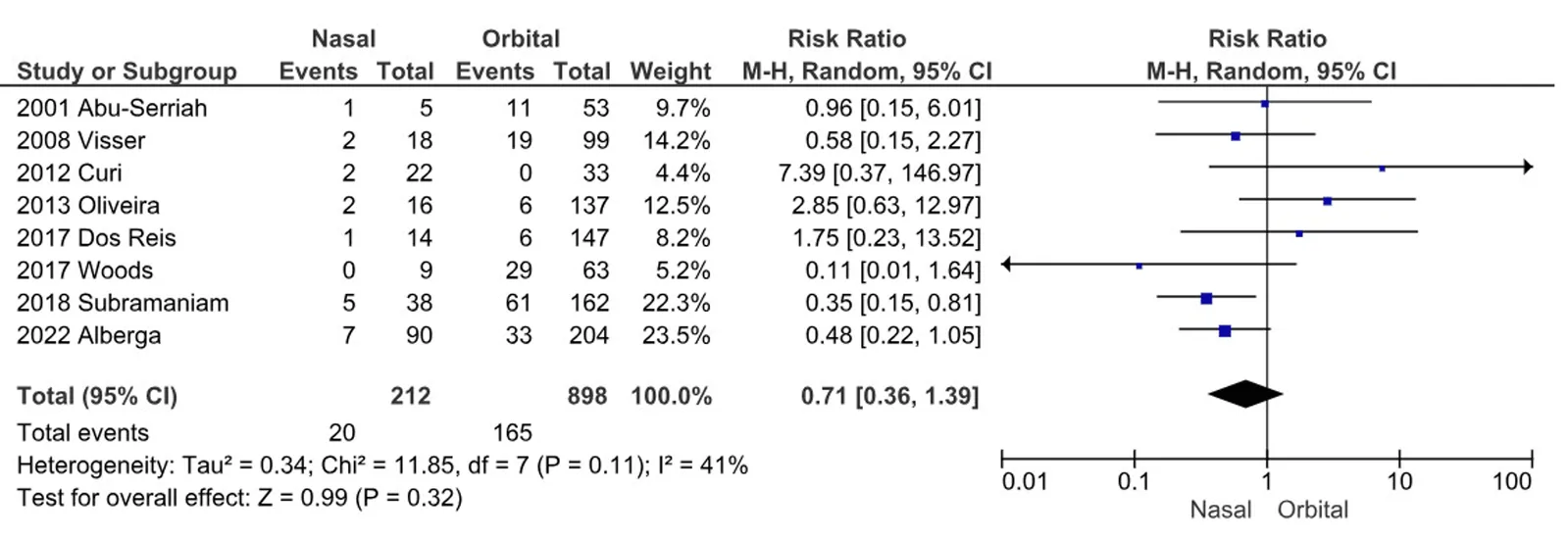
Forest plot showing the comparison of implant failure risk between nasal and orbital craniofacial implants Source: [10,15,17,18,20-23]

Radiotherapy Meta‑Analysis

Five studies [10,15,18,21,22] showed irradiated patients had a significantly higher risk of implant failure than non‑irradiated patients (RR = 1.99; 95% CI: 1.09-3.66; p = 0.03; I² = 45%) (Figure 5). However, details regarding radiation dose, timing, and clinical indications were inconsistently reported, and several studies had small sample sizes with wide confidence intervals, indicating imprecision that should be acknowledged when interpreting pooled results.

**Figure 5:**
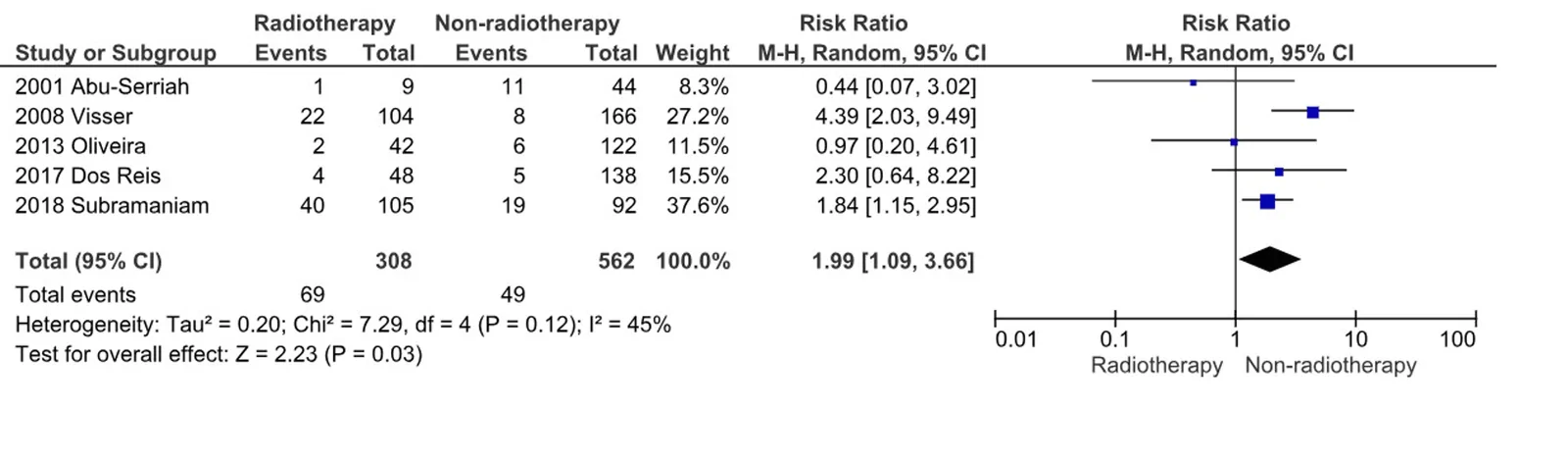
Forest plot showing the comparison of implant failure risk between irradiated and non-irradiated patients receiving extra-oral craniofacial implants Source: [10,15,18,21,22]

Summary

Overall, both anatomical site and radiotherapy status significantly influenced outcomes. Auricular implants had the most favorable survival, orbital implants carried higher failure risk, and radiotherapy increased implant loss. Meta‑analysis results are summarized in Table 4.

**Table 4:** Summary of the quantitative meta-analysis results

Comparison	Studies (n)	Experimental Events/Total	Control Events/Total	Risk Ratio (95% CI)	P-value	I² (%)
Orbital vs Auricular implants	8	165/898	52/749	2.64 (1.32-5.30)	0.006	70
Nasal vs Auricular implants	8	20/212	52/749	1.77 (0.83-3.80)	0.14	35
Nasal vs Orbital implants	8	20/212	165/898	0.71 (0.36-1.39)	0.32	41
Irradiated vs Non-irradiated patients	5	69/308	49/562	1.99 (1.09-3.66)	0.03	45

Discussion

This systematic review and meta-analysis synthesizes survival outcomes of extraoral craniofacial implants, with a particular focus on auricular, orbital, and nasal sites, while examining the impact of radiotherapy. Unlike earlier reviews that pooled heterogeneous implant sites, our analysis highlights clinically important variations according to anatomical location and radiation exposure. Auricular implants demonstrated significantly higher survival compared to orbital implants, whereas no significant differences were observed between nasal and auricular implants or between nasal and orbital implants. Radiotherapy was consistently associated with an increased risk of implant failure.

Auricular implants yielded the most favorable results, as reported by previous studies [10,11,15,17,20,23]. These outcomes are likely explained by the dense cortical bone, robust vascularity, and favorable biomechanics of the temporal bone. In contrast, orbital implants showed lower survival rates, which can be attributed to reduced bone volume, compromised vascularity, prior surgical resections, and frequent exposure to radiotherapy [17,18,22,23]. Nasal implants generally demonstrated intermediate survival, though the available data were limited. Midfacial implants were excluded from quantitative analysis due to variability in defect configuration and inconsistent reporting, but qualitative evaluation suggested lower and more unpredictable survival, particularly in reconstructed and irradiated defects [16,17,19].

Radiotherapy remains a critical determinant of implant outcome. Its biological effects-reduced blood supply, hypocellularity, fibrosis, and impaired bone remodeling-directly hinder osseointegration [24,25]. Patients who received radiation had a significantly greater risk of implant failure, consistent with these biological mechanisms. Adjunctive treatments such as hyperbaric oxygen may improve outcomes, as reported by Granström [13,25]. Variations among studies likely reflect differences in radiation dose, timing, implant design, and follow-up duration [13,17,18,22,23,28-31].

Ureel et al. confirmed these findings, noting site-specific variations and poorer outcomes among irradiated patients [32]. They emphasized the importance of long-term follow-up and multidisciplinary care, which aligns with the conclusions of the present review. Reported complications included peri-implant soft tissue reactions, inflammation, granulation tissue, prosthetic maintenance requirements, and implant loss, particularly in orbital rehabilitation [11-23]. Most of these complications were manageable with routine care.

From a clinical perspective, implant site should be a key determinant in treatment planning. Auricular implants offer the most predictable survival, while orbital implants require careful patient selection and long-term monitoring. Multidisciplinary strategies, as emphasized by Korfage et al. [33], are essential for successful rehabilitation. Beyond survival rates, outcomes also depend on prosthetic maintenance, aesthetics, patient expectations, and quality of life [34].

Statistically, the use of a random-effects model was justified to account for expected variability across studies in terms of patient populations, surgical techniques, and radiation protocols. Nevertheless, interpretation of pooled estimates must remain cautious given the limited number of included studies. The substantial heterogeneity observed in the orbital versus auricular comparison (I² = 70%) reflects differences in study design, radiation exposure, and follow-up duration. Sensitivity analyses confirmed the robustness of the main findings, but also underscored the need for standardized reporting in future research.

Overall, auricular implants represent the most favorable site for implant-retained prostheses. Orbital implants carry a higher risk of failure, and radiotherapy significantly increases implant loss, underscoring the need for individualized planning, patient counseling, and multidisciplinary follow-up. Most included studies were retrospective, introducing potential bias. Heterogeneity reflected differences in study design, populations, implant sites, radiotherapy protocols, and follow-up. Lack of prospective protocol registration reduced transparency. Comparison with recent reviews highlights consistent findings, though publication bias remains possible. Future prospective multicenter studies with standardized reporting of survival, complications, radiation dose, and patient-reported outcomes are essential to strengthen the evidence base.

## Conclusions

Within the limitations of the available evidence, extra-oral craniofacial implant survival is influenced by both anatomical site and radiotherapy status. Auricular implants demonstrated the most favourable survival outcomes and significantly superior performance compared with orbital implants, whereas radiotherapy was associated with a significantly increased risk of implant failure. However, as most included studies were retrospective and observational, these findings should be interpreted cautiously. These findings provide clinically relevant evidence to guide treatment planning, patient counselling, and long‑term maintenance strategies in craniofacial implant rehabilitation.

## Appendices

Complete search strategies

1. PubMed/MEDLINE

("extraoral implants"[MeSH Terms] OR "craniofacial implants"[All Fields] OR "facial implants"[All Fields])

AND ("implant survival"[All Fields] OR "implant failure"[All Fields] OR "complications"[All Fields])

AND ("auricular implants"[All Fields] OR "orbital implants"[All Fields] OR "nasal implants"[All Fields])

AND ("radiotherapy"[MeSH Terms] OR "irradiated patients"[All Fields])

Filters: Humans, English, Publication date from 2000/01/01 to 2025/12/31

2. Scopus

TITLE-ABS-KEY("extraoral implants" OR "craniofacial implants" OR "facial implants")

AND TITLE-ABS-KEY("implant survival" OR "implant failure" OR "complications")

AND TITLE-ABS-KEY("auricular implants" OR "orbital implants" OR "nasal implants")

AND TITLE-ABS-KEY("radiotherapy" OR "irradiated patients")

AND (LIMIT-TO(LANGUAGE, "English")) AND (PUBYEAR > 1999 AND PUBYEAR < 2026)

3. Web of Science

TS=("extraoral implants" OR "craniofacial implants" OR "facial implants")

AND TS=("implant survival" OR "implant failure" OR "complications")

AND TS=("auricular implants" OR "orbital implants" OR "nasal implants")

AND TS=("radiotherapy" OR "irradiated patients")

Refined by: Document Types=(Article) AND Languages=(English) AND Timespan=2000-2025

4. Cochrane Library

("extraoral implants" OR "craniofacial implants" OR "facial implants")

AND ("implant survival" OR "implant failure" OR "complications")

AND ("auricular implants" OR "orbital implants" OR "nasal implants")

AND ("radiotherapy" OR "irradiated patients")

Limits applied: Humans, English, 2000-2025
